# 3D printing of Ceffe-infused scaffolds for tailored nipple-like cartilage development

**DOI:** 10.1186/s12896-024-00848-3

**Published:** 2024-04-30

**Authors:** Jinghao Ding, Chuanzhi Wei, Yong Xu, Wufei Dai, Ru Chen

**Affiliations:** 1 Department of Breast Surgery, Hainan Affiliated Hospital of Hainan Medical University (Hainan General Hospital), Haikou, China; 2 Department of Plastic and Reconstructive Surgery, Shanghai Ninth People’s Hospital, Shanghai Jiao Tong University School of Medicine, Shanghai, China; 3 Department of Thoracic Surgery, Shanghai Pulmonary Hospital, Tongji University School of Medicine, Shanghai, China

**Keywords:** Nipple reconstruction, 3D printing, Ceffe, Tissue engineering, Vascularization

## Abstract

The reconstruction of a stable, nipple-shaped cartilage graft that precisely matches the natural nipple in shape and size on the contralateral side is a clinical challenge. While 3D printing technology can efficiently and accurately manufacture customized complex structures, it faces limitations due to inadequate blood supply, which hampers the stability of nipple-shaped cartilage grafts produced using this technology. To address this issue, we employed a biodegradable biomaterial, Poly(lactic-co-glycolic acid) (PLGA), loaded with Cell-Free Fat Extract (Ceffe). Ceffe has demonstrated the ability to promote angiogenesis and cell proliferation, making it an ideal bio-ink for bioprinting precise nipple-shaped cartilage grafts. We utilized the Ceffe/PLGA scaffold to create a porous structure with a precise nipple shape. This scaffold exhibited favorable porosity and pore size, ensuring stable shape maintenance and satisfactory biomechanical properties. Importantly, it could release Ceffe in a sustained manner. Our in vitro results confirmed the scaffold’s good biocompatibility and its ability to promote angiogenesis, as evidenced by supporting chondrocyte proliferation and endothelial cell migration and tube formation. Furthermore, after 8 weeks of in vivo culture, the Ceffe/PLGA scaffold seeded with chondrocytes regenerated into a cartilage support structure with a precise nipple shape. Compared to the pure PLGA group, the Ceffe/PLGA scaffold showed remarkable vascular formation, highlighting the beneficial effects of Ceffe. These findings suggest that our designed Ceffe/PLGA scaffold with a nipple shape represents a promising strategy for precise nipple-shaped cartilage regeneration, laying a foundation for subsequent nipple reconstruction.

## Introduction

The perfect breast requires well-proportioned nipples as the finishing touch. With the increasing incidence of breast cancer, the number of patients undergoing mastectomy resulting in nipple loss is growing. Other causes such as congenital absence, inflammation, and trauma can also lead to nipple deformity or absence, affecting not only the physical image of women but also causing psychological stress [1, 2]. Nipple reconstruction has long been one of the challenges faced by plastic and breast surgeons. Nipple reconstruction is an essential component of breast reconstruction. Reconstructing aesthetically pleasing and symmetric nipples can increase patient satisfaction with the overall breast reconstruction and alleviate psychological barriers [3, 4]. To achieve this goal, numerous reconstruction techniques have been proposed for nipple reconstruction, including local flap, free tissue transplantation, and local flap combined with autologous or allogeneic transplantation [5, 6]. However, these methods have limitations in replicating a nipple support structure that matches the shape and size of a natural nipple completely. For example, using the local flap method for nipple reconstruction, the unstable vascularization of the flap and scar contracture make it difficult to predict the final appearance of the nipple [6, 7]. Although using autologous rib cartilage for nipple reconstruction slightly improves nipple protrusion, carving a nipple cartilage support structure that precisely matches the anatomical structure of the natural nipple is challenging [8–10]. Nipple sharing requires patients to sacrifice a healthy natural nipple in exchange for two incomplete and inferior nipples, and there are potential complications such as impaired breastfeeding ability, sensory abnormalities, and nipple deformities in the donor nipple [5]. Complications of synthetic materials such as silicone rods and artificial bones are relatively high, with nipple necrosis and infection making it difficult to maintain nipple shape [11, 12]. Therefore, there is an urgent need to seek a more effective and safer strategy for precise nipple support reconstruction.

In recent years, the emergence of tissue engineering technology has provided a new approach for repairing tissue defects. Tissue engineering scaffolds are solid or gel-based support structures that, when seeded with cells, can serve as tissue grafts, providing the necessary volume and space for tissue regeneration. 3D printing technology, widely used in the biomedical field, has become an advanced auxiliary technique for tissue engineering and regenerative medicine [13–15]. Compared to tissue engineering scaffolds produced by traditional techniques (gas foaming, solvent casting, freeze-drying, electrospinning, etc.), 3D-printed cartilage scaffolds have unique advantages in scaffold personalization, accuracy, and spatial structural complexity [16]. Current research has found that 3D-printed cartilage tissue engineering scaffolds have advantages in high porosity, uniform pore distribution, and compressive strength [17]. Tissue engineering scaffolds provide support for cell infiltration and proliferation and create favorable space for the regeneration and reconstruction of the extracellular matrix. Therefore, tissue engineering scaffolds printed using 3D printing technology not only have a fine internal three-dimensional porous structure, which is conducive to cell adhesion and proliferation, but more importantly, the scaffold’s shape can precisely match the anatomical structure of the defective tissue [18, 19].

Adequate blood supply is crucial for cartilage survival. From the outset of implantation in the body, tissue-engineered scaffolds seeded with cartilage cells require sufficient nutrient supply, especially in the central region, because nutrient supply by diffusion is limited to tissues within 150–200 μm thickness [20, 21]. Without blood vessels to ensure adequate nutrient supply, cell death in the center of the scaffold is inevitable, leading to loss of scaffold shape and function. The main reason may be that the lack of nutrients leads to apoptosis of cartilage cells and insufficient secretion of extracellular matrix by cartilage cells. Inability to maintain the original size and vascularization of cartilage implants within the scaffold is a major obstacle in cartilage tissue engineering [22]. Therefore, a cartilage scaffold graft that promotes angiogenesis is key to improving cartilage survival and thereby enhancing the shape-maintaining ability of nipple-shaped cartilage support structures.

Ceffe is a liquid extract of fat tissue obtained rapidly by pure physical methods [23]. Ceffe contains a large amount of VEGF, EGF, and other growth factors, which can promote angiogenesis and cell proliferation. It has been applied to promotes tendon repair, enhance angiogenesis in limb ischemia, improve ischemic flap survival, and promote cartilage regeneration [23–26]. Ceffe is abundant, easy to obtain, and free of cellular components, making it low in immunogenicity and safe. Currently, there is no research on using biological materials loaded with Ceffe to prepare tissue engineering scaffolds suitable for 3D printing to reconstruct nipple cartilage support structures.

PLGA is a copolymer of glycolic acid and lactic acid in different ratios, with good biocompatibility, no tissue reaction in the body, and good degradation properties. In this study, we prepared Ceffe-loaded PLGA composite biomaterials and based on it for 3D printing, to prepare cartilage regeneration scaffolds with precise nipple shapes. Utilizing the slow degradation characteristics of PLGA, Ceffe-loaded PLGA scaffolds can slowly release Ceffe to promote angiogenesis, thereby providing necessary nutrient supply for cartilage regeneration and ultimately achieving the regeneration of cartilage support structures with precise nipple shapes. In addition, we studied the possibility of implanting Ceffe/PLGA scaffolds seeded with cartilage cells to generate cartilage supports with precise nipple shapes in vivo using a nude mouse subcutaneous model.

## Materials and methods

### Extraction of Ceffe

The experiment received approval from the Ethics Committee of Hainan Affiliated Hospital, Hainan Medical University. Ceffe was extracted from fresh adipose tissue using a previously established method [25]. The conventional liposuction technique was employed to extract abdominal fat from six healthy female New Zealand White Rabbits (2 months old, weighing approximately 2.5 kg, Shanghai Yunde Biotechnology Co., Ltd, China). The freshly extracted adipose tissue underwent a process to remove excess blood cells, followed by centrifugation at 1200 rpm for 3 min. This step discarded the upper lipid and lower liquid layers, retaining the middle fat layer, which was then mechanically emulsified. The emulsified fat was frozen at -80 °C and subsequently thawed at 37 °C. After thawing, it underwent further centrifugation at 1200 g for 5 min at 4 °C. Post-centrifugation, the third liquid layer (Ceffe) was extracted, passed through a 0.22 μm filter to eliminate bacteria and cell debris, and stored at -20 °C for later use.

### 3D Printing of PLGA and Ceffe/PLGA scaffolds

1 g of PLGA crystals (Sigma, USA) was dissolved in 10 mL of dichloromethane-acetone organic solvent (8 ml dichloromethane and 2 ml acetone, Sigma, USA). The solution underwent vortex shaking three times for 15 s each, followed by centrifugation at 1000 rpm for 30 s to form a 10% w/v PLGA solution. Additionally, 5 g of polyvinyl alcohol dissolved in 50 mL phosphate buffer saline (PBS, Sigma, USA) solution. The PLGA solution was mixed with the polyvinyl alcohol solution, supplemented with 1 mg of Ceffe (designated as Ceffe/PLGA group) or without Ceffe (designated as PLGA group). The mixture was placed in a thermostatic stirrer and stirred overnight. Subsequently, the mixture underwent shock emulsification through an ultrasonic breaker for 5 min and stirring in a magnetic stirrer for 3 h. Finally, the mixture was centrifuged at 1000 rpm for 10 min to remove the organic solvent, resulting in the formation of Ceffe/PLGA and PLGA colloidal solutions.

Customized brackets, nipple-shaped in design, were created using computer-aided design through Magics software (Materialise Corporation). The scaffold design incorporated a porous structure (300 μm pore size for micropores) to facilitate nutrient diffusion and surrounding tissue growth. The Ceffe/PLGA and PLGA colloidal solutions were then 3D printed on a 3D printer (GeSim BioScaffolder, Germany) to obtain nipple-shaped Ceffe/PLGA and PLGA scaffolds. 3D printing parameter settings: nozzle temperature 25℃; platform cooling temperature 3℃; printing speed 2 mm/s; line spacing 0.5 mm. The scaffold specifications are 10 mm in diameter at the base and 5 mm in height.

### Characterizations of PLGA and Ceffe/PLGA scaffolds

#### Morphological and pore size observation

The macromorphology of PLGA and Ceffe/PLGA scaffolds was observed using a digital camera (Nikon, Japan). Additionally, the micromorphology of the two scaffolds was assessed via scanning electron microscopy (SEM, S3400, Hitachi, Japan) at an acceleration voltage of 10 kV for microstructural inspection. ImageJ software was utilized for further analysis of the average pore size based on the SEM images.

#### Porosity determination

Porosity of PLGA and Ceffe/PLGA scaffolds was determined using a liquid replacement method. The original volume of ethanol was denoted as V_1_, the volume of the scaffold after 5 min of immersion in ethanol as V_2_, and the residual volume after removing the scaffold as V_3_. The porosity of scaffold was calculated using the formula: (V_1_-V_3_)/(V_2_-V_3_).

#### Shape maintenance

After immersion in PBS for 4 weeks, PLGA and Ceffe/PLGA scaffolds were photographed immediately upon removal from wells for area determination. The projected area of the scaffolds was assessed by ImageJ based on the images.

#### Mechanical strength

Mechanical strength of the scaffolds was determined using a mechanical testing machine. PLGA and Ceffe/PLGA scaffolds underwent continuous planar unconfined strain at a rate of 1 mm/min until 80% of maximal deformation was achieved. Young’s modulus was calculated according to the stress-strain curve.

#### Degradation rate

Dry PLGA and Ceffe/PLGA scaffolds were initially weighed as W_1_ and then immersed in sterile PBS (pH = 7.4) at 37 °C with continuous shaking. Scaffolds were retrieved at predetermined times (1, 2, 3, 4, 5, 6, 8, 10, 12 weeks), lyophilized, and weighed as W_2_. The degradation rate was calculated using the formula: W_2_/W_1_ × 100%.

#### Release kinetics of Ceffe from Ceffe/PLGA scaffold

The in vitro release kinetics of Ceffe from Ceffe/PLGA scaffolds were investigated by immersing the scaffolds in deionized water at 37 °C. The release medium was extracted at specific time intervals, replaced with an equal volume of deionized water. Ceffe concentration in the collected solution was determined by a BCA kit ^[2]^ at predetermined times (1, 2, 3, 4, 5, 6, 8, 10, 12 weeks), and Ceffe release percentage was calculated.

### Isolation and culture of chondrocytes

All animal procedures received approval from the Ethics Committee of Hainan Affiliated Hospital, Hainan Medical University. Auricular cartilage was harvested from the aforementioned New Zealand White Rabbits, immersed in an antibiotic solution for 30 min, minced into approximately 0.5–2.0 mm³ pieces, and digested with 0.15% type II collagenase (Sigma, USA) in Dulbecco’s Modified Eagle’s Medium (DMEM, Gibco, USA) for 8 h at 37 °C to isolate chondrocytes. The isolated chondrocytes were cultured in DMEM supplemented with 10% fetal bovine serum (FBS, Gibco, USA) and 1% double antibody (Gibco, USA) at 37 °C in 5% CO_2_. Chondrocytes were collected at the second passage for use.

### In vitro biocompatibility

#### Cell proliferation assay

To assess the biocompatibility of the PLGA and Ceffe/PLGA scaffolds, chondrocytes were resuspended in DMEM containing 10% FBS, and the cell density was adjusted to 1.0 × 10^6^ cells/mL before seeding onto PLGA and Ceffe/PLGA scaffolds. Cells were cultivated in vitro for 5 days in DMEM at 37 °C in 5% CO_2_. The viability of chondrocytes directly seeded on scaffolds on days 1, 3, and 5 was evaluated using a live/dead cell viability assay (Sigma, USA). Live/dead staining images were observed under a laser scanning confocal microscope (CLSM, Leica, Germany). Cell proliferation was measured using a cell counting kit-8 (CCK-8, Sigma, USA) according to the manufacturer’s instructions, with optical density (OD) measured at 450 nm.

#### Cell morphology

The morphology of chondrocytes cultured in vitro within PLGA and Ceffe/PLGA scaffolds was observed with CLSM. Samples collected on days 1, 3, and 5 were fixed in 4% paraformaldehyde for 30 min, washed three times with PBS, permeabilized with 0.1% Triton X-100 (Sigma, USA) for 30 min, and then incubated with Phalloidin-iFluor for 30 min. After further washing, the chondrocytes were stained with 4’,6-diamidino-2-phenylindole (DAPI, Sigma, USA) for 10 min.

### Angiogenic evaluation

#### Extraction of scaffolds

PLGA and Ceffe/PLGA scaffolds were disinfected with a 75% ethanol solution for 60 min, washed twice with PBS, and then placed into DMEM. The solution was incubated for 24 h at 37 °C, collected, filtered with a 0.22 μm filter, and stored at 4 °C for long-term preservation.

#### Migration of human umbilical vein endothelial cells (HUVECs)

1.0 × 10^5^ HUVECs, obtained from the Type Culture Collection of the Chinese Academy of Sciences (Shanghai, China), were seeded in the upper chamber of transwell 24-well plates. PLGA and Ceffe/PLGA scaffold extracts were added to the lower chamber of the transwell. After 4 and 12 h, the upper chambers were removed, and the cells on the lower chamber were stained with 0.1% crystal violet. HUVEC counts per field were analyzed using ImageJ software.

#### Capillary formation assay

HUVECs were used for capillary formation assays. MatrigelTM was prepared overnight at 4°C, with 50 µL added per well of a chilled 96-well plate. HUVECs and extracts of PLGA and Ceffe/PLGA scaffolds were subsequently added (3 × 10^4^ cells/well), followed by incubation for 4 and 12 h at 37 °C. Tube formation in both groups was observed via a light microscope, and quantification for tubes and branch points per field were measured using Image J software.

### In vivo nipple-shaped cartilage regeneration

#### Preparation of chondrocyte-scaffold construct

PLGA and Ceffe/PLGA scaffolds were sterilized in a 75% ethanol solution overnight, followed by three washes with sterile saline solution. Chondrocytes at the 2nd passage, with a density of 1.0 × 10^8^ cells/mL, were then separately packed into PLGA and Ceffe/PLGA scaffolds, forming chondrocyte-scaffold constructs.

#### Subcutaneous implantation of chondrocyte-scaffold construct

Six nude mice (4 weeks old, weighing approximately 200 g, Shanghai Yunde Biotechnology Co., Ltd, China) were anesthetized using 1% sodium pentobarbital. The backs were sterilized, and a skin incision was made, creating a pocket in the subcutaneous tissue. The constructs in both PLGA and Ceffe/PLGA groups were implanted into the pocket, the incision was closed, and the mice were incubated for 2 and 8 weeks. Samples were collected from the backs of nude mice for gross observation and sectioned for histological evaluation.

#### Histological evaluation

After gross observation using a digital camera, samples were fixed in 4% buffered formalin for 24 h, embedded in paraffin, and cut into 5 μm sections. One part of the sections was used for histological staining, and the other for multiple immunofluorescence (mHIC) staining. Sections were stained with hematoxylin & eosin (HE) and Safranin-O to evaluate the histological structure and cartilage-specific extracellular matrix (ECM) deposition in the regenerated tissue.

#### Biochemical analysis

Specimens from nude mice were digested in papain solution at 65 °C. The sulfated glycosaminoglycan (GAG) content of samples in PLGA and Ceffe/PLGA groups was quantified by Alcian Blue. The samples were hydrolyzed at 100 °C with HCl, and hydroxyproline content was determined using the Hydroxyproline Assay Kit. In addition, collagen type II (COL II) content was quantified using Enzyme-linked Immunosorbent Assay.

#### mHIC staining

The sections were fixed with 4% paraformaldehyde for 30 min, permeabilized in PBS containing 1% Triton X-100 for 10 min, followed by washing three times with PBS, and blocked with 10% goat serum in PBS for 30 min. Then, the sections were incubated with primary antibodies against CD31 (abcam, UK) or collagen type II (abmart, China) in the Superblock solution overnight at 4 °C. On the next day, sections were washed with PBS three times for 5 min each, followed by incubation with the Goat Anti-Rabbit IgG (abcam, UK) or Goat Anti-Rat IgG H&L (abcam, UK) for 2 h at room temperature. Nuclei were counterstained with 4,6-diamidino-2-phenylindole (DAPI) for 2 min. After washing, the tissue was observed under the CLSM in the darkroom. Quantification for CD31 and COL II intensities were determined based on the obtained mHIC images via ImageJ software.

### Statistical analysis

All numerical data were presented as mean ± standard deviation. Differences between two groups were analyzed using Student’s t-test, and differences between multiple groups were analyzed using one-way analysis of variance (ANOVA) followed by Tukey’s post-hoc test. All analyses were performed using SPSS 22.0 software (IBM SPSS, Chicago, IL, USA). Differences were considered statistically significant at *P* < 0.05.

## Results

### Characterizations of 3D printed PLGA and Ceffe/PLGA scaffolds

**Fig. 1:**
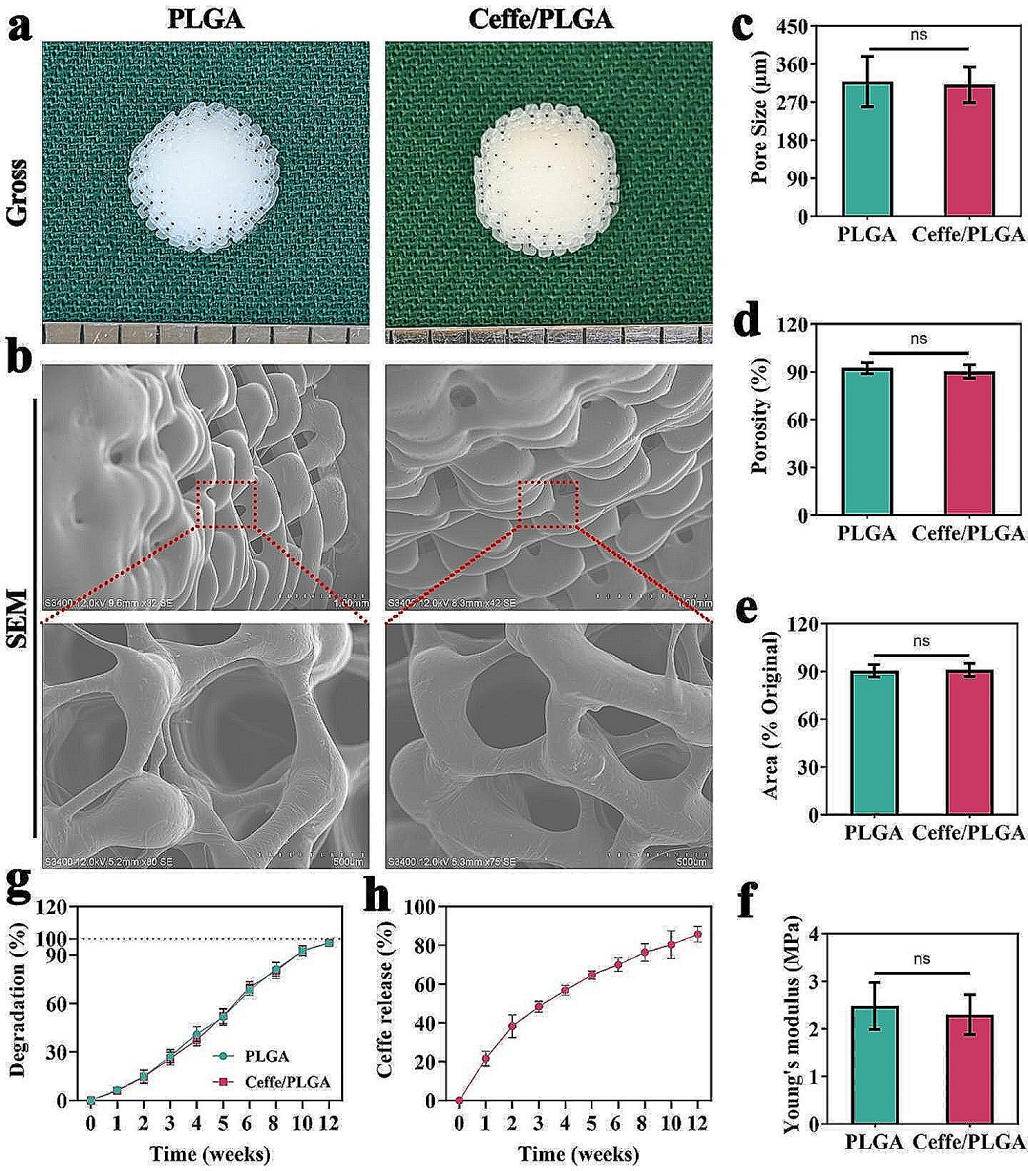
Characterizations of 3D printed Ceffe/PLGA scaffold with nipple shape. **a**) Gross observation of PLGA and Ceffe/PLGA scaffolds. **b**) SEM observation of PLGA and Ceffe/PLGA scaffolds. **c**) Pore size of PLGA and Ceffe/PLGA scaffolds. **d**) Porosity of PLGA and Ceffe/PLGA scaffolds. **e**) The projected area of PLGA and Ceffe/PLGA scaffolds after immersion in PBS for 4 weeks. **f**) Young’s modulus of PLGA and Ceffe/PLGA scaffolds. **g**) Degradation of PLGA and Ceffe/PLGA scaffolds after immersion in PBS for 12 weeks (pH = 7.4). **h**) Cumulative Ceffe release from Ceffe/PLGA scaffolds after immersion in PBS for 12 weeks (pH = 7.4). “ns”, no statistical significance

In this study, PLGA and Ceffe/PLGA scaffolds were prepared via 3D printing. Gross images revealed that both scaffolds had nipple-shaped trapezoidal columnar structures. The PLGA scaffold appeared white, while the Ceffe/PLGA scaffold appeared slightly red due to the addition of Ceffe (Fig. 1a). SEM images demonstrated that both PLGA and Ceffe/PLGA scaffolds displayed a similar three-dimensional porous structure (Fig. 1b). Quantitative analyses indicated that the pore sizes of PLGA and Ceffe/PLGA scaffolds were 318.4 ± 53.2 μm and 311.0 ± 37.6 μm, respectively, and the porosities were 92.4 ± 3.1% and 90.3 ± 3.8%, respectively (Fig. 1c-d). Scaffolds submerged in PBS for 4 weeks exhibited only slight contraction, suggesting that the addition of Ceffe did not affect the anti-contraction capability of the scaffold (Fig. 1e). Quantitative analyses of Young’s modulus (Fig. 1f) and degradation (Fig. 1g) revealed that Ceffe had an insignificant effect on the mechanical strength and degradation rate of the scaffolds. The release curve indicated that Ceffe was almost completely released by the 12th week, aligning with the degradation rate of Ceffe/PLGA scaffolds (Fig. 1h), suggesting that the release rate of Ceffe was related to the degradability of Ceffe/PLGA scaffolds.

### Biocompatibility evaluation

**Fig. 2:**
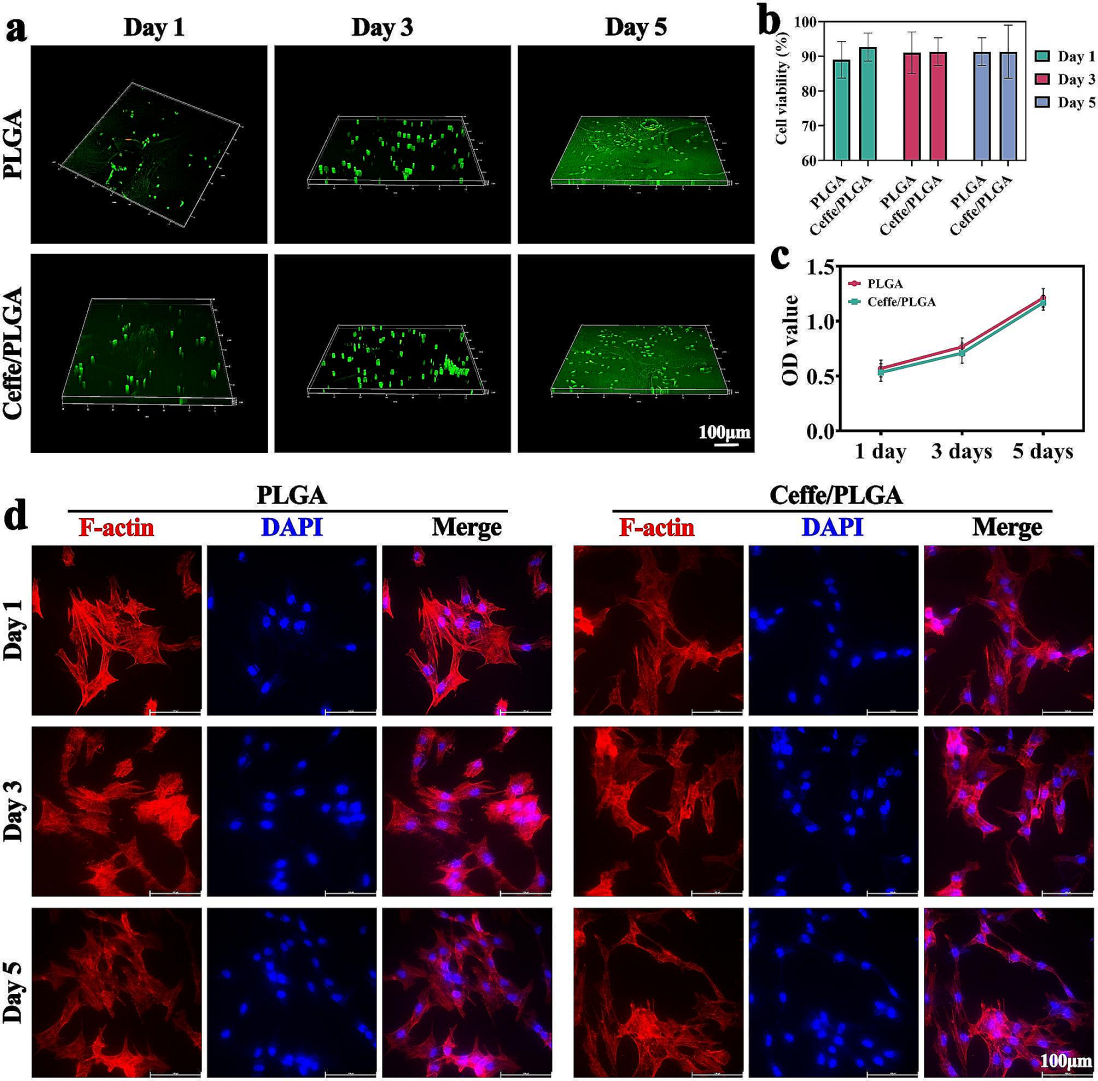
Biocompatibility of Ceffe/PLGA scaffold. **a**) Live/dead staining of chondrocyte-loaded PLGA and Ceffe/PLGA scaffolds at 1, 3, and 5 days. Quantification of **b**) cell viability and **c**) OD value of chondrocyte-loaded PLGA and Ceffe/PLGA scaffolds at 1, 3, and 5 days. **d**) F-actin/DAPI staining of chondrocyte-loaded PLGA and Ceffe/PLGA scaffolds at 1, 3, and 5 days, in which red stained F-actin denote cytoskeleton and bule stained DAPI denote nucleus

Biocompatibility is a crucial feature of tissue engineering scaffolds. Fluorescence images of live/dead cell staining showed that chondrocytes inoculated in both PLGA and Ceffe/PLGA scaffolds were viable (stained in green), with only a few dead cells detected (stained in red) (Fig. 2a). Quantitative analysis of cell viability showed that cells maintained a high level of viability with extended incubation time (Fig. 2b). Moreover, OD values determined by the CCK-8 assay revealed that chondrocytes in the PLGA and Ceffe/PLGA groups showed similar levels and a marked increasing trend (Fig. 2c). F-actin/DAPI staining showed that cells adequately covered both PLGA and Ceffe/PLGA surfaces (Fig. 2d). Cells exhibited a polygonal shape, and the cytoskeleton of cells was observed. The results indicated that both scaffolds were biocompatible, suggesting that the addition of Ceffe had no effect on the cytocompatibility of the Ceffe/PLGA scaffolds in vitro.

### Angiogenic assessment

**Fig. 3:**
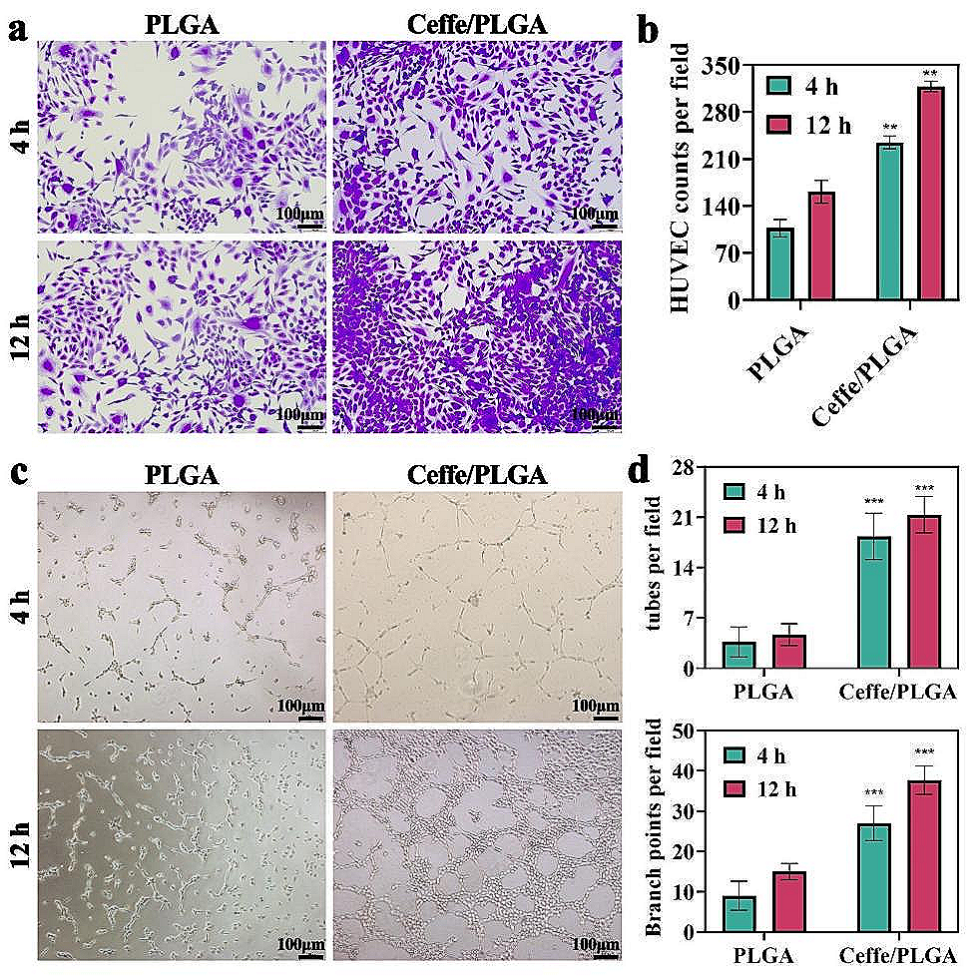
In vitro pro-angiogenic evaluation of Ceffe/PLGA scaffold. **a**) Crystal violet staining of HUVECs when cocultured with PLGA and Ceffe/PLGA scaffolds at 4 and 12 h. **b**) Quantification of HUVEC counts in PLGA and Ceffe/PLGA groups at 4 and 12 h. **c**) Light microscope of tube formation for HUVECs when cocultured PLGA and Ceffe/PLGA groups at 4 and 12 h. **d**) Quantification of branch points and tubes per filed in PLGA and Ceffe/PLGA groups at 4 and 12 h. **, *P* < 0.01. ***, *P* < 0.001

To evaluate the pro-angiogenic effects, HUVECs were co-cultured with PLGA and Ceffe/PLGA scaffolds, and the functions of migration and tube formation in HUVECs were measured. Transwell assays assessing cellular migration determined that the migration capabilities of HUVECs in the Ceffe/PLGA group were significantly stronger than those in the PLGA group (Fig. 3a-b). The results of the tube formation assay showed that Ceffe/PLGA significantly enhanced the tube formation of HUVECs (Fig. 3c), as confirmed through the quantification of numbers of tubes and branch points (Fig. 3d).

### In vivo nipple-shaped cartilage regeneration

**Fig. 4:**
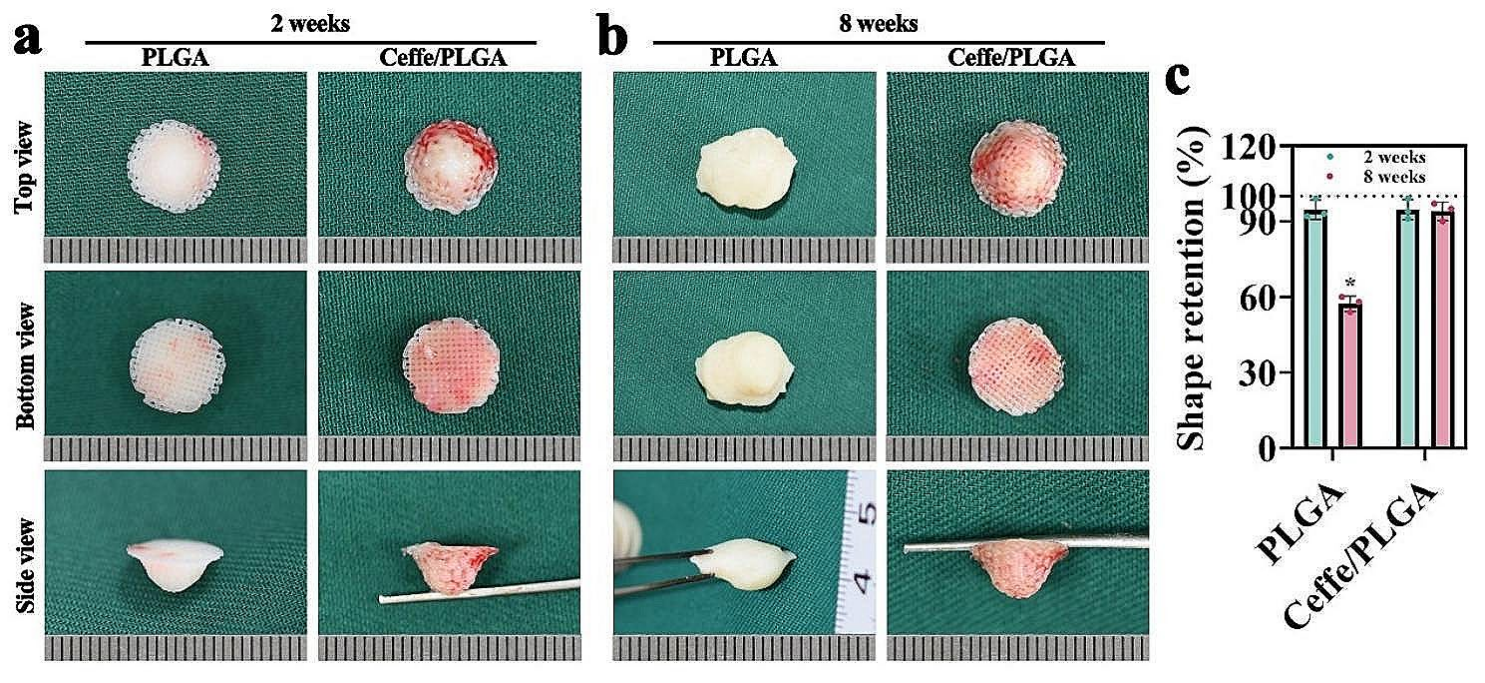
In vivo nipple shaped cartilage regeneration. **a**) Gross observation in top, bottom, and side views of nipple shaped cartilage regeneration in PLGA and Ceffe/PLGA groups after 2 weeks subcutaneously implantation in nude mice. **b**) Gross observation in top, bottom, and side views of nipple shaped cartilage regeneration in PLGA and Ceffe/PLGA groups after 8 weeks subcutaneously implantation in nude mice. **c**) Quantification of shape retention rate in PLGA and Ceffe/PLGA groups after 2 and 8 weeks subcutaneously implantation in nude mice. *, *P* < 0.05

Both PLGA and Ceffe/PLGA scaffolds filled with chondrocytes were respectively implanted subcutaneously on the backs of nude mice for 2 and 8 weeks to verify the possibility of precise development of nipple-shaped cartilage supports in animals. After 2 weeks of implantation, gross observation showed that samples in both the PLGA group and the Ceffe/PLGA group retrieved from nude mice exhibited white cartilage-like tissue, with the whole sample displaying a nipple-like appearance (Fig. 4a-b). Notably, a small amount of neovascularization was observed on the surface of the cartilage tissue in the Ceffe/PLGA group. At 8 weeks post-implantation, gross observation showed that samples in both groups from nude rats showed a more typical cartilage-like appearance. Moreover, samples in the Ceffe/PLGA group were encapsulated within a thin layer of vascularized tissue. However, in comparison to the PLGA group, samples from the Ceffe/PLGA group still maintained a nipple-like appearance. Quantitative analysis of shape retention rate further confirmed that samples in the Ceffe/PLGA group had significantly better shape retention than those in the PLGA group at 8 weeks post-implantation (Fig. 4c).

### Histological and immunohistochemical analysis

**Fig. 5:**
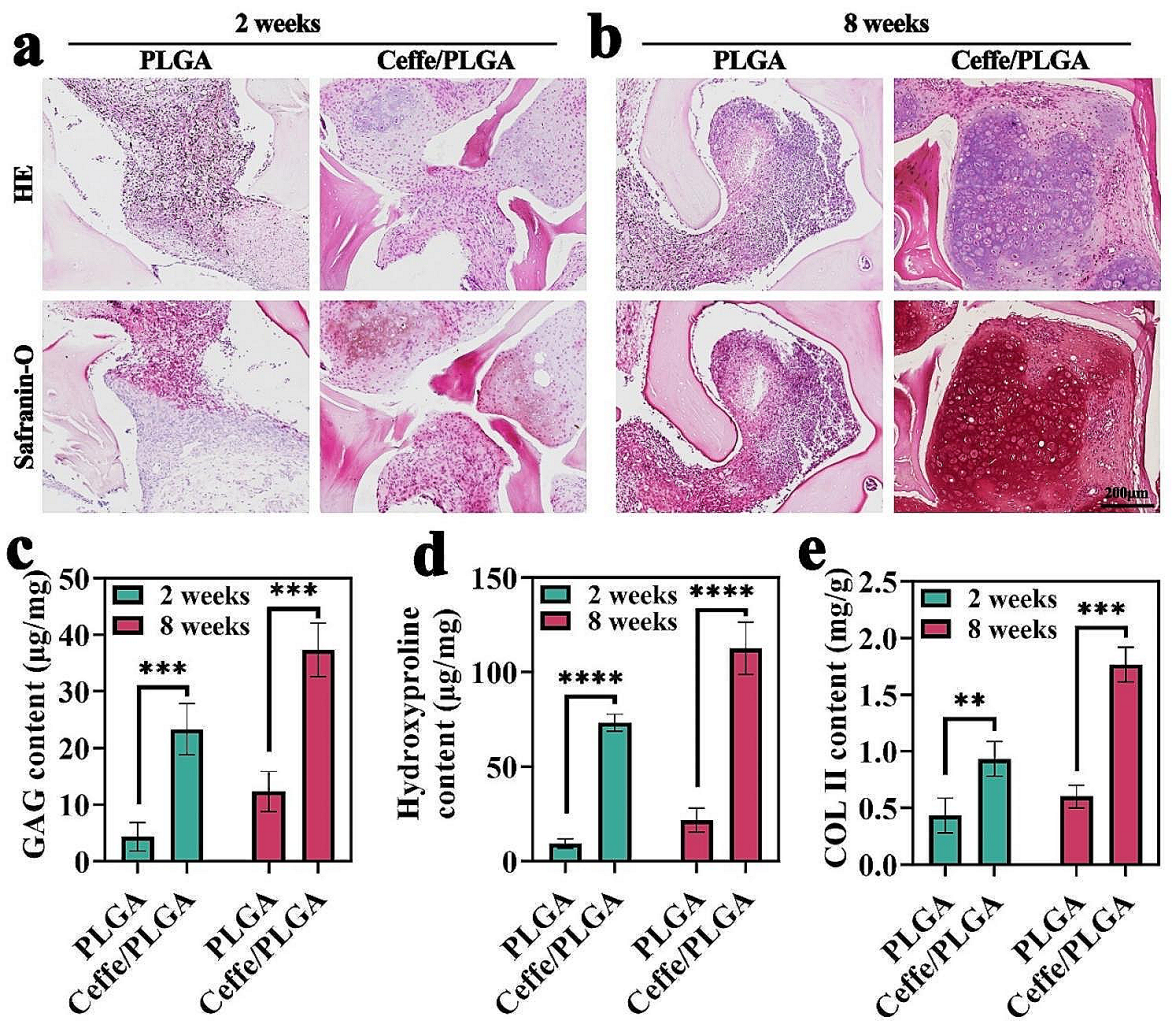
Histological evaluation of in vivo-engineered nipple shaped cartilage. **a**) HE and Safranin-O stainings of samples in PLGA and Ceffe/PLGA groups at 2 weeks. **b**) HE and Safranin-O stainings of samples in PLGA and Ceffe/PLGA groups at 8 weeks. Quantification of **c**) GAG, **d**) hydroxyproline, and **e**) COL II contents in PLGA and Ceffe/PLGA groups at 2 and 8 weeks. ***, *P* < 0.001. ****, *P* < 0.0001

After 2 weeks of implantation, histological analyses showed that the sample in the Ceffe/PLGA group exhibited cartilage-specific ECM deposition and typical lacunar structures. In contrast, samples in the PLGA group displayed a chimeric structure comprised of both typical cartilage-specific ECM and fibrous-like features (Fig. 5a). At 8 weeks post-implantation, histological analyses revealed that samples in the Ceffe/PLGA group showed a more typical cartilage-like appearance than those in the PLGA group. In contrast, the sample in the PLGA groups showed apparently less intensive cartilage-specific staining than the Ceffe/PLGA group (Fig. 5b). Quantitative analysis showed that with the prolongation of in vivo culture time, the GAG, hydroxyproline, COL II contents in the Ceffe/PLGA group exhibited an increasing trend, surpassing those in the PLGA group (Fig. 5c-e).

**Fig. 6:**
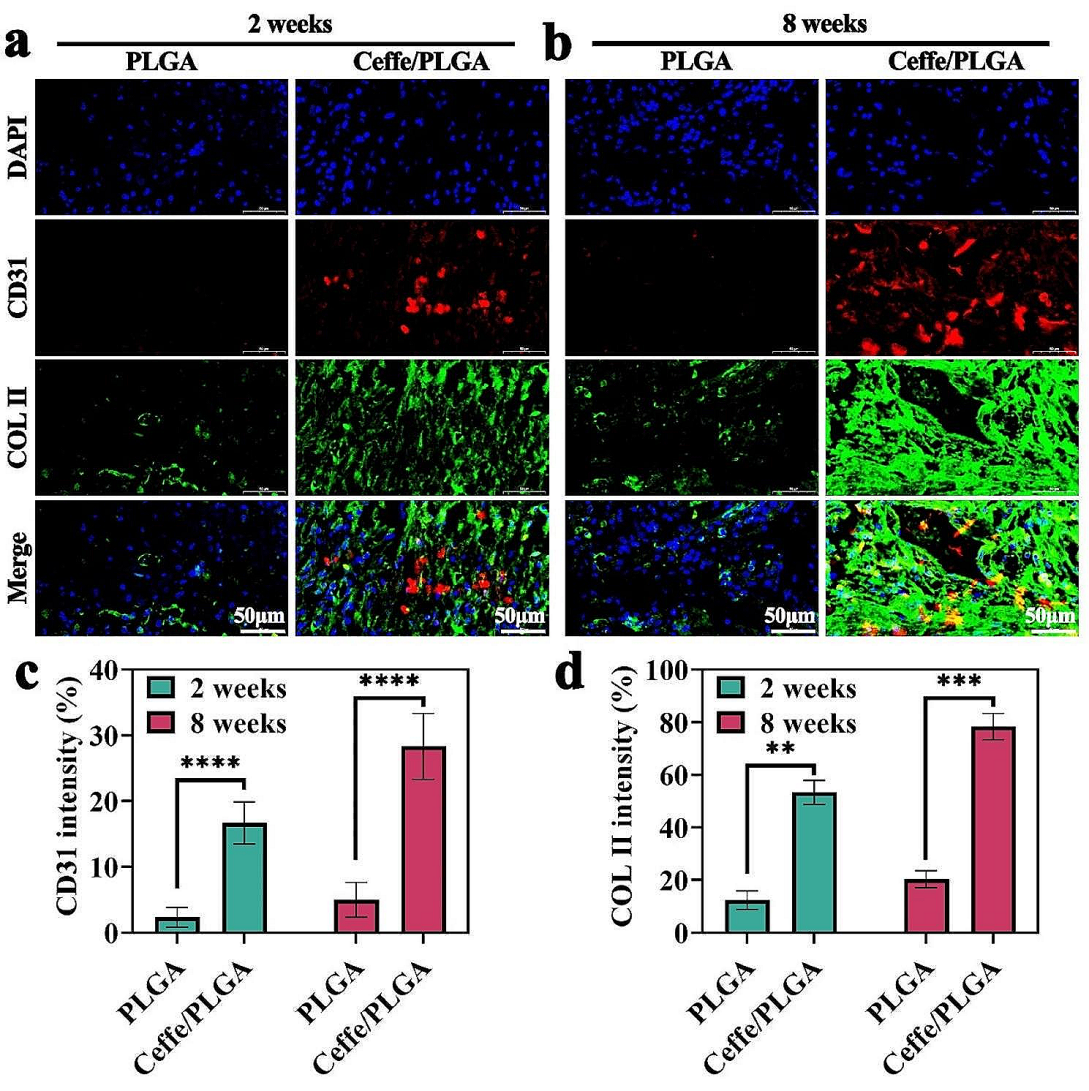
Immunofluorescence assessment of in vivo-engineered nipple shaped cartilage. **a**) Immunofluorescence CD31 and COL II stainings of samples in PLGA and Ceffe/PLGA groups at 2 weeks. **b**) Immunofluorescence CD31 and COL II stainings of samples in PLGA and Ceffe/PLGA groups at 8 weeks. Quantification of **c**) CD31 and **d**) COL II intensities in PLGA and Ceffe/PLGA groups at 2 and 8 weeks. **, *P* < 0.01. ***, *P* < 0.001. ****, *P* < 0.0001

This trend was consistent with histological staining results, where the expression levels of CD31 and COL II via mHIC staining in the PLGA groups were lower than those in the Ceffe/PLGA group at both 2 and 8 weeks (Fig. 6a-b). Notably, a more intense positive staining for CD31 and COL II was observed in the Ceffe/PLGA group at 8 weeks compared to 2 weeks.

Quantitative analysis showed that with the prolongation of in vivo culture time, the intensities of CD31 and COL II in the Ceffe/PLGA group showed an increasing trend, outperforming those in the PLGA group (Fig. 6c-d). These results indicated that the Ceffe/PLGA scaffolds represent an ideal scaffold for cartilage regeneration in vivo, and that Ceffe was favorable for vessel formation and cartilage-specific ECM production.

## Discussion

Reconstructing a cartilage scaffold with an accurately replicated nipple shape is an international challenge. The main challenge in this field is to prepare a cartilage support that is the same shape and size as the natural nipple and to maintain its shape in vivo. Currently, this study has prepared a Ceffe/PLGA scaffold using 3D printing technology, seeded with cartilage cells, and then implanted it into the body to reconstruct the nipple cartilage support. The nipple cartilage support reconstructed in vivo has the same precise shape as the natural nipple.

Nipple reconstruction is the final step in breast reconstruction after mastectomy. Areola reconstruction can be performed using medical tattooing, a simple procedure that accurately reproduces the appearance and color of the reconstructed areola based on the opposite areola, thus avoiding the need to design this tissue [27]. Therefore, the focus of the study is on nipple reconstruction. Currently, many nipple reconstruction techniques have been developed. Compared to simple local flap reconstruction of the nipple, using autologous or allogeneic transplants as nipple supports can maintain nipple protrusion and shape for a longer period of time. Surgeons provide additional support within the local flap using autologous or allogeneic tissues or synthetic materials, such as cartilage, acellular dermal matrix (ADM), hyaluronic acid, artificial bone, and silicone. Hwang et al. designed a semilunar skin flap combined with an Omega-shaped acellular dermal matrix for nipple reconstruction, and six months postoperatively, most patients maintained a nipple protrusion rate of more than 60% [28]. However, using ADM as an internal support within the flap cannot shape a cartilage support that matches the opposite nipple precisely, and the high cost of ADM limits the promotion of this technology. Sue et al. injected hyaluronic acid filler into the nipple base of 12 patients who underwent local flap reconstruction of the nipple and experienced a decrease in nipple protrusion. The average volume of filler used per nipple was 0.35 ml, and the average increase in nipple protrusion was 3.0 mm. The nipples remained stable six months postoperatively, but the nipples injected with filler did not maintain good symmetry with the opposite nipple in appearance, and as hyaluronic acid degraded, the nipple protrusion would decrease again, requiring repeated injections of filler [29]. Yanaga used Ceratite implement (artificial bone) as a nipple support within the flap to maintain nipple shape and protrusion. All 100 patients followed up postoperatively maintained nipple protrusion, but the complication rate reached 18%, including flap necrosis (5%), partial skin necrosis at the graft site (8%), and exposure of the artificial bone (5%) [30]. Heitland et al. reported using autologous rib cartilage to carve a nipple support combined with an arrow-shaped flap for nipple reconstruction. The average decrease in nipple protrusion rate at 6-month follow-up was 24.87%. However, using a whole piece of rib cartilage to carve a nipple support greatly tests the surgeon’s carving ability and sense of three-dimensionality [9, 10, 31]. In summary, current nipple reconstruction techniques cannot reproduce a perfect nipple that precisely matches the shape of the opposite nipple. Although the nipple protrusion retention rate is the primary concern of all nipple reconstruction techniques, the aesthetics of nipple reconstruction and the symmetry of the appearance of both nipples also need to be paid attention to by surgeons, which is also related to patient satisfaction with overall nipple reconstruction.

3D printing technology allows for the precise, rapid, and reproducible manufacture of complex structures. By scanning the contralateral nipple and using bio-synthetic materials as printing ink, it is possible to prepare a nipple support structure that matches the shape of the healthy nipple of the patient. Artificially synthesized biodegradable materials, due to their rigidity, low immunogenicity, and degradability, are used for tissue engineering scaffolds printed in 3D. PLGA is a biodegradable copolymer with biocompatibility approved by the Food and Drug Administration or European Medicines Agency for some biomedical applications [32]. It is composed of lactide and glycolide portions, and its molecular weight and composition ratio can actively influence drug release and enzyme degradation rates, preventing rapid drug release in the body. PLGA undergoes hydrolysis through lactide and glycolide ester bonds in the body. Then, these monomers can be metabolized through the tricarboxylic acid cycle, producing non-toxic by-products (H_2_O and CO), which are beneficial for the application of PLGA in the medical field [33]. Shu et al. used 3D printing technology to prepare cobalt-incorporated chloroapatite/PLGA scaffolds, which were implanted into a joint cartilage defect model and promoted cartilage repair through antioxidant and anti-inflammatory effects [34].

In this study, the printing parameters were investigated for their potential effects on the activity of bioactive factors in Ceffe. Firstly, the printing temperature was room temperature, and the platform cooling temperature was 3 °C. Despite the low cooling temperature, Ceffe can be stored long-term at -20 °C to -80 °C. Thus, theoretically, the printing parameters should have no effect on Ceffe. Secondly, drug release kinetics experiments on the scaffold detected proteins in Ceffe. Results from biocompatibility assessments and in vitro angiogenesis evaluations indicate that scaffolds with added Ceffe promote cell proliferation and angiogenesis. Finally, results from in vivo animal experiments demonstrate that the histological examination, biochemical analysis, and immunofluorescence data of the Ceffe/PLGA group samples are superior to those of the PLGA group samples. Therefore, we conclude that 3D printing has not significantly impacted the bioactivity of Ceffe.

It has been reported that using grafts as support materials within the flap can provide a longer-lasting nipple projection and resistance to scar contracture. However, the most common complication when using these materials is flap or graft necrosis. Common causes of flap necrosis are inadequate blood supply and excessive pressure stimulation. Additionally, adequate blood supply is also required for cartilage regeneration. Studies have reported using adipose tissue derivatives to promote cartilage regeneration, such as adipose-derived stem cells (ADSCs) and stromal vascular fraction (SVF) [35]. Adipose tissue derivatives are derived from mature adipose tissue, with SVF containing ADSCs, endothelial progenitor cells, and hematopoietic stem cells, among others. Experimental studies have shown that both ADSCs and SVF can secrete vascular endothelial growth factors and differentiate into endothelial cells to participate in angiogenesis. However, the immunogenicity and potential tumorigenicity of stem cells limit their application [36, 37]. Ceffe is a cell-free liquid that can be easily prepared without the need for cell culture, thus avoiding safety issues related to cell therapy. In addition, Ceffe is non-immunogenic and non-tumorigenic, and can potentially be used not only for autologous but also for allogeneic sources [26]. Through proteomic analysis, Ceffe has been found to contain a large number of biologically active proteins, including vascular endothelial growth factors, which have a good angiogenic effect. Cai et al. found that by injecting Ceffe into a rat flap ischemia model, it could increase flap survival by promoting the number of capillaries in the ischemic flap [25]. Jia et al. demonstrated that Ceffe containing factors similar to those produced by stem cells can promote the growth of chondrocytes and exert anti-inflammatory effects by inhibiting the M0 to M1 polarization of macrophages [26].

In this study, we prepared two groups of scaffolds: PLGA scaffolds and Ceffe/PLGA scaffolds. SEM observations showed that both groups exhibited relatively uniform three-dimensional porous structures. Quantitative analysis revealed that the pore size of the PLGA group was 318.4 ± 53.2 μm with a porosity of 92.4 ± 3.1%, while the pore size of the Ceffe/PLGA group was 311.0 ± 37.6 μm with a porosity of 90.3 ± 3.8%. These structures provide space for chondrocyte proliferation, extracellular matrix secretion, and new capillary formation. Previous studies have shown that growth factors in Ceffe degrade rapidly and are unstable in vivo [38]. Therefore, in this study, by loading Ceffe into PLGA scaffolds and utilizing the slow degradation characteristics of PLGA, Ceffe is gradually released to improve its utilization. As expected, as the Ceffe/PLGA scaffold gradually degrades, Ceffe is slowly released, allowing the growth factors and cytokines in Ceffe to continuously exert their effects on promoting angiogenesis and cell proliferation. Biocompatibility evaluation is crucial to determine if the scaffolds are suitable for cartilage regeneration. Our results showed that both groups of scaffolds exhibited good cell viability and low cytotoxicity, promoting chondrocyte proliferation. Furthermore, the angiogenic effect of Ceffe was confirmed by its ability to promote endothelial cell migration and tube formation in vitro.

In subcutaneous cartilage regeneration experiments in nude mice, the Ceffe/PLGA scaffold group showed superior ability to promote cartilage regeneration and formation compared to the PLGA scaffold group. As the in vivo cultivation time extended, fibrovascular tissue formed on the surface of the cartilage grafts, providing nutrition supply for chondrocyte proliferation and extracellular matrix secretion, supporting the generation and maintenance of nipple-shaped cartilage support [39]. Histological staining and immunofluorescence staining further confirmed this. Safranin-O is a specific stain for observing cartilage tissue, showing a higher positive staining for chondrocytes in the Ceffe/PLGA group compared to the PLGA scaffold group. Quantitative analysis showed that the content of glycosaminoglycan (GAG) and hydroxyproline in the Ceffe/PLGA scaffold group was significantly higher than in the PLGA group. GAG and hydroxyproline are the main components of collagen, which is the main component of the extracellular matrix. The extracellular matrix not only provides structural support for chondrocytes but also contains bioactive signals known to regulate cell adhesion, proliferation, and differentiation, playing an important role in cartilage formation. CD31 is an endothelial-specific immunohistochemical marker for blood vessels, showing that the degree of vascularization in the Ceffe/PLGA group was significantly higher than in the PLGA scaffold group. COL II is a specific immunohistochemical marker for chondrocyte extracellular matrix, showing that the secretion of extracellular matrix by chondrocytes in the Ceffe/PLGA group was significantly higher than in the PLGA scaffold group. These results indicate that the ability of the Ceffe-loaded PLGA scaffold to promote the formation of nipple-shaped cartilage supports is closely related to its enhanced angiogenesis and cell proliferation abilities. In contrast, the PLGA scaffold, lacking the ability to promote angiogenesis and cell proliferation, did not ultimately form nipple-shaped cartilage supports in in vivo cultivation. The potential mechanism is insufficient blood supply, leading to chondrocyte apoptosis and inadequate extracellular matrix secretion. Therefore, we believe that the Ceffe/PLGA scaffold has great potential in shaping nipple-shaped cartilage supports.

## Conclusion

In conclusion, we have successfully prepared a Ceffe/PLGA scaffold with chondrogenic capability and demonstrated its ability to support chondrocyte proliferation in vitro and regenerate nipple-shaped cartilage supports in vivo. However, the limitations of this study include the use of only nude mice (immunodeficient animals) for in vivo experiments and a relatively short observation period (8 weeks). Additionally, we did not explore the composition ratio of PLGA and the concentration of Ceffe. Therefore, our next step will be to further investigate the composition ratio of the Ceffe/PLGA scaffold to optimize its angiogenic and chondrogenic effects. We also plan to assess the feasibility of regenerating nipple-shaped cartilage supports in rabbits or large animals (such as goats) to provide experimental evidence for the clinical application of the Ceffe/PLGA scaffold in humans.

## Data Availability

The datasets generated during and/or analyzed during the current study are available from the corresponding author (R.C.) on reasonable request.
